# Making Sense out of Antisense Transcription in Human T-Cell Lymphotropic Viruses (HTLVs)

**DOI:** 10.3390/v3050456

**Published:** 2011-05-05

**Authors:** Benoit Barbeau, Jean-Michel Mesnard

**Affiliations:** 1 Département des Sciences Biologiques, Centre de recherche Bio Med, Université du Québec à Montréal, Montréal (Québec) H2X 3X8, Canada; E-Mail: barbeau.benoit@uqam.ca; 2 Centre d’études d’agents Pathogènes et Biotechnologies pour la Santé, Université Montpellier 1, 34293 Montpellier Cedex 5, France; 3 CNRS, UM5236, CPBS, F-34965 Montpellier, France; 4 CPBS, Université Montpellier 2, F-34095 Montpellier, France

**Keywords:** HTLV, HBZ, antisense transcription, LTR, chronic infection

## Abstract

Retroviral gene expression generally depends on a full-length transcript that initiates in the 5′ long terminal repeat (LTR), which is either unspliced or alternatively spliced. We and others have demonstrated the existence of an antisense transcript initiating in the 3′ LTR of the Human T-cell Leukemia Virus type 1 (HTLV-1) that is involved in the production of HBZ (HTLV-1 basic leucine zipper (bZIP) factor). HBZ is a Fos-like factor capable of inhibiting Tax-mediated activation of the HTLV-1 LTR by interacting with the cellular transcription factor cAMP-response element-binding protein (CREB) and the pleiotropic cellular coactivators p300/CBP. HBZ can also activate cellular transcription through its interaction with p300/CBP. Interestingly, HBZ has also been found to promote T-lymphocyte proliferation. By down-regulating viral expression and by stimulating T-cell proliferation, HBZ could be essential in the establishment of a chronic infection. Antisense transcription also occurs in the closely related HTLV-2 retrovirus as well as in the recently discovered HTLV-3 and HTLV-4. These antisense transcripts are also involved in the production of retroviral proteins that we have termed Antisense Protein of HTLVs (APH). Like HBZ, the APH proteins are localized in the nucleus of transfected cells and repress Tax-mediated viral transcription.

## 1. Introduction

Human T-cell leukemia virus type 1 (HTLV-1) is a complex lymphotropic retrovirus classified in the Deltaretrovirus genus of the retrovirus family. Other members include HTLV-2, -3, and -4, the simian T-cell lymphotropic viruses (STLV-1, -2, and -3) and the bovine leukemia virus. HTLV-1 is associated with adult T-cell leukemia (ATL) and a slowly progressive neurologic disorder, HTLV-1 associated myelopathy/tropical spastic paraparesis (HAM/TSP). ATL develops after a prolonged latency period of up to 30–50 years postinfection and is an aggressive lymphoproliferative disease with different clinical stages considered to gradually progress from carrier to smoldering, chronic, and acute-type leukemia [1]. Leukemic cells in ATL are almost exclusive CD4^+^ T cells. HAM/TSP has a shorter incubation period than ATL and can show an accelerated development within one month to four years after transfusion of the infected blood sample. The main pathologic features of HAM/TSP are chronic inflammation in the spinal cord, characterized by perivascular lymphocytic cuffing and parenchymal lymphotropic infiltration including HTLV-1-infected CD4^+^ T cells [2]. Unlike human immunodeficiency virus type 1, HTLV-1 causes no disease in a majority of infected subjects. Approximately 2% to 3% develop ATL and another 2% to 3% develop a disabling chronic inflammatory disease involving the central nervous system (HAM/TSP), eyes, lungs, or skeletal muscles.

**Figure 1:**
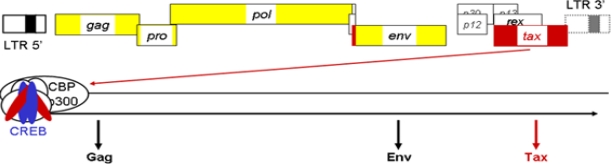
Schematic representation of the Human T-cell Leukemia Virus type 1 (HTLV-1) proviral genome. In addition to the common retroviral genes (in yellow), the provirus contains genes encoding different regulatory proteins. Among them, the Tax protein (in red) interacts with CREB (in blue) to bind to the Tax-responsive elements (TxREs). Tax then stabilizes the TxRE-bound complex and recruits p300/CBP to stimulate the viral transcription and the production of the different viral proteins such as Gag, Env, and Tax.

As with most retroviruses, HTLV-1 begins its cycle with the infection of target cells. Following viral entry, the viral RNA genome is reverse transcribed into a double-stranded DNA molecule and enters the nucleus as a nucleic acid-protein complex, which mediates the integration of proviral DNA into the host chromatin. The proviral DNA possesses two long terminal repeats, the 5′ and 3′ LTRs, composed of three regions, U3, R, and U5. The U3 region of the LTR contains important elements like the Tax-responsive elements (TxREs). The viral Tax protein does not bind specifically to the TxREs but interacts with some members of the activating transcription factor/CRE-binding (ATF/CREB) proteins such as CREB and CREB-2 [3,4] that are able to bind to TxRE DNA regions (Figure 1). The formation of such a complex on the 5′ LTR then serves as a binding site for the recruitment of the pleiotropic cellular coactivators p300/CBP through its interaction with Tax. Recruitment of p300/CBP to the viral promoter induces local nuclesosome modification by histone acetylation and facilitates stable binding of components of the basal transcription machinery allowing the stimulation of viral transcription [5] and ensuing synthesis of the viral proteins including Gag, Env, and Tax (Figure 1). The production of these viral proteins induces an immune response toward HTLV-1 [6]. The first specific antibodies to appear are directed against Gag and are predominant in the first two months. Subsequently, anti-Env and anti-Tax antibodies appear. In addition, the majority of HTLV-1-specific CD8^+^ T cells recognize the Tax protein.

**Figure 2:**
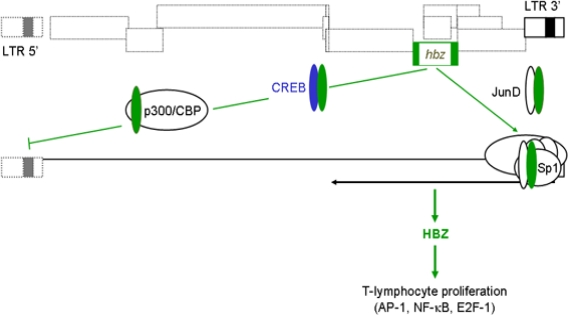
Regulation of sense and antisense transcription by HTLV-1 basic leucine zipper (bZIP) factor (HBZ). Antisense transcripts initiated from the 3′ LTR are responsible for encoding the HBZ protein. HBZ then downmodulates Tax-dependent viral gene expression by interacting with CREB and p300/CBP. HBZ could also activate its own expression by forming HBZ/JunD heterodimers able to interact with the Sp1 transcription factor bound to the Sp1-binding sites involved in the stimulation of antisense transcription. HBZ would thus render the infected cells less likely to be targeted by the immune response through lower expression of viral proteins but should also promote T-lymphocyte proliferation by controlling cellular transcription through AP-1, NF-κB and E2F-1 pathways.

All retroviral genes have been thought to be transcribed through a single promoter located in the 5′ LTR of the provirus. However, the presence of a conserved open reading frame (ORF) in the complementary strand of the HTLV-1 provirus suggested the existence of viral mRNA of negative polarity. The existence of antisense transcription in HTLV-1 was demonstrated for the first time in 1989 through Northern blot analysis of RNA extracted from an HTLV-1-infected cell line [7]. However, it is only 13 years later that we provided the first evidence of a protein termed HTLV-1 basic leucine zipper (bZIP) factor (HBZ) produced from an HTLV-1 antisense transcript [8]. This finding led to a number of studies aimed at examining the transcript itself. The initial positioning of the *hbz* gene showed that it was located between the *env* gene and the last exon of the *tax* transcript (Figure 2). RACE experiments have revealed that transcription initiation sites were all located in the 3′ LTR, precisely in the R and U5 regions [9]. Importantly, these experiments showed that the antisense transcript was spliced and produced a major spliced form with the ATG initiation codon located in exon 1 in the 3′ LTR segment. These results were also confirmed by other teams using different approaches [10,11]. Focus was also given to the identification of the 3′ end of the transcript. In agreement with the initial suggested polyA signal, we and others have confirmed its usage for 3′ processing of the HBZ transcript and addition of the polyA tail [9,11].

## 2. HBZ Is a c-Fos-Like Factor

HBZ is nuclear factor containing a transcriptional activation domain at its N-terminus and a ZIP domain at its C-terminus [8,12,13]. Different isoforms have been described, sharing about 95% amino acid sequence identity differing only at their N termini [9,10,14]. The most abundant HBZ form detected in HTLV-1-infected cell lines corresponds to the 206 amino acid-long isoform produced from the major spliced variant. This messenger can be detected in numerous infected cell lines and directly in cells isolated from infected patients [9–11,15–18]. HBZ interacts with c-Jun and JunB through its ZIP domain [19,20]. This interaction leads to a reduction in c-Jun and JunB DNA-binding activity and prevents these proteins from activating transcription of AP-1-dependent promoters by sequestering them into nuclear bodies [21,22], corresponding to transcriptionally inactive sites. It has also been proposed that HBZ inhibits c-Jun activity by promoting its degradation through a proteasome-dependent pathway [20].

### 2.1. HBZ Down-Regulates the Viral Sense Transcription but Stimulates the Antisense Transcription

Early studies demonstrated that the ZIP domain of HBZ was also able to interact with CREB-2 [8], a member of the ATF/CREB family that is involved in Tax-dependent activation of the viral transcription [23]. This interaction blocks the binding of CREB-2 to the HTLV-1 LTR and thereby abolishes Tax-dependent activation of promoter activity [8,24]. These preliminary data were confirmed through standard chromatin immunoprecipitation experiments, which highlighted the displacement of CREB from the HTLV-1 LTR upon HBZ expression [25]. In addition, HBZ harbors an N-terminal activation domain that contains two LXXLL-like motifs. These motifs mediate direct binding of HBZ to the cellular coactivators p300/CBP [26], which specifically occurs through the KIX domain that is conserved between the coactivators. p300 and CBP play central roles in activation of HTLV-1, as well as cellular transcription by serving as scaffolds for other transcriptional regulators to associate with promoters and through their histone acetyltransferase activity. In the context of HTLV-1 transcription, HBZ effectively displaces p300/CBP from the viral promoter. This mechanism appears to be more potent than that of the ZIP domain in mediating repression of viral transcription (Figure 2).

HBZ also interacts with JunD [27], the third member of the Jun family. HBZ does not inhibit JunD activity unlike c-Jun and JunB. Indeed, HBZ is able to cooperate with JunD to enhance transcription by interacting with the Sp1 transcription factor. In these conditions, activation of transcription by this protein complex is mediated through binding sites for Sp1 present in the promoter [28]. Interestingly, Sp1 sites have been described to be involved in the regulation of antisense transcription from the 3′ LTR [14,29]. Thus, HBZ could not only negatively control expression of the other viral proteins to avoid deleterious immune response but would also be able to stimulate its own expression.

### 2.2. HBZ Controls Cellular Transcription

Additional HBZ interaction partners of HBZ have been characterized. By conducting a yeast two-hybrid assay using HBZ, MafB was identified as a partner [30]. HBZ heterodimerizes with MafB via its ZIP domain. However, the role of this interaction remains unclear. It has been proposed that HBZ has a suppressive effect on Maf function [30], but when MafB was tested in the presence of HBZ in a gel-shift assay using a Maf recognition elements, the HBZ/MafB heterodimer directly bound DNA [31]. DNA binding was specific and is dependent both on the HBZ basic region and on DNA that flanked the central binding site. Because the sequence of the HBZ basic region is unique, it may have a distinct DNA-binding specificity and it remains possible that cellular promoters could be recognized *in vivo* by HBZ in association with Maf proteins.

HBZ also stimulates JunD transcriptional activity [27]. By interacting with JunD, HBZ forms a complex with stronger accessibility to transcriptional factors or cofactors bound to cellular promoters [13]. Hence, the HBZ-JunD heterodimer is then able to cooperate with the Sp1 transcriptional factor to enhance *hTERT* transcription through Sp1-binding sites present in the proximal sequences of the promoter [28]. In addition to its ZIP domain, the activation domain of HBZ appears to be essential for up-regulating the *hTERT* promoter activity. Recently, expression of a protein involved in bone resorption, Dkk1, has also been demonstrated to be activated by HBZ through its interaction with p300/CBP [32]. However, this effect of HBZ is limited in HTLV-1-infected T-cell lines, which in part may be due to suppression of Dkk1 expression by Tax. Consequently, the ability of HBZ to regulate expression of Dkk1 and possibly other cellular genes may only be significant following loss of Tax expression, which is an event frequently observed during progression of ATL [33].

HBZ has also been found to bind the NF-κB subunit p65 [34]. This interaction results in an inhibition of the classical NF-κB activation pathway. Both activation and ZIP domains are involved in the binding of HBZ to p65. By interacting with p65, HBZ inhibits DNA binding of p65 like for c-Jun and JunB. However, HBZ is also able to increase expression of the E3 ubiquitin ligase, PDLIM2, resulting in ubiquitination and degradation of p65 [34]. PDLIM2 has also been described to suppress Tax-mediated tumorigenesis by recruiting Tax from its functional sites into the nuclear matrix where Tax is degraded by the proteasome [35]. Recently, immunoprecipitation experiments showed that HBZ also interacted with both Foxp3 and NFAT, interrupting the function of both cellular factors [36].

## 3. HBZ Promotes T-Lymphocyte Proliferation

In addition to its capacity to disrupt AP-1 and NF-κB pathways, observations favored a possible impact of HBZ on ATL development, specifically on ATL maintenance. In ATL cells, the occurrence of 5′ LTR methylation or its deletion is frequent, thereby leading to the inhibition of viral gene expression including Tax. In addition, as opposed to the 5′ LTR, the 3′ LTR is hypomethylated [37]. Moreover, as mentioned above, HBZ could stimulate its own expression. Taken together, these observations explain why HBZ expression is consistently detected in ATL cells [11]. It could be speculated that Tax might rather be involved in the first step of transformation while HBZ would act as a maintenance factor in ATL cells. Indeed, shRNA repression of *hbz* gene expression in established HTLV-1-transformed cell lines and newly immortalized T lymphocytes significantly suppressed T-lymphocyte proliferation [38]. Very recent results demonstrate that *hbz* gene induced T-cell lymphoma in transgenic mice [36] but the long latent period before the onset of lymphoma in these mice confirms that additional events are necessary in addition of *hbz* expression. Moreover, the HBZ RNA itself could also promote T-cell proliferation [11]. This model would therefore argue for a possible bimodal function of HBZ whereby the protein could act upon different cell function/viral gene expression while the transcript would positively enhance cell proliferation. Microarray analysis indicated that the HBZ RNA was responsible for the upregulation of *E2F-1* [11].

## 4. Regulation of the Balance between Sense and Antisense Transcription

**Figure 3:**
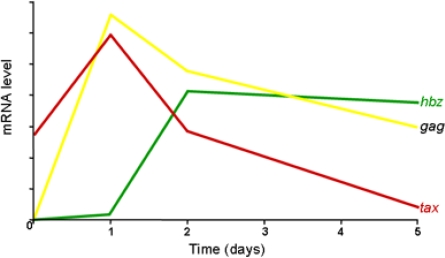
Kinetic analysis of HTLV-1 transcription. Quantification of *tax* (red), *gag* (yellow) and *hbz* (green) mRNAs in lymphocytes from HTLV-1 associated myelopathy/tropical spastic paraparesis (HAM/TSP) patients following *ex vivo* culture.

CD4^+^ T cells freshly isolated from HAM/TSP patients spontaneously expressed HTLV-1 sense transcripts from the 5′ LTR, initially Tax transcript then Gag RNA (Figure 3). This expression reached a peak after about 24 h of incubation. With respect to antisense transcription from the 3′ LTR, HBZ RNA expression increased after two days of culture and then plateaued to stable levels [39]. It is interesting to notice that increase of antisense transcription from the 3′ LTR corresponds to a decrease of sense expression from 5′ LTR suggesting that the loss of sense transcription results in increased antisense transcription. We have already observed such results by analyzing sense/antisense transcription from 293T cells transfected with either a full-length proviral clone or a molecular clone without its 5′ LTR [9,40]. The absence of the 5′ LTR stimulated the synthesis of antisense transcripts from the 3′ LTR. It might be postulated that sense and antisense transcription could compete for a limited amount of common cellular transcription factors involved in the formation of the preinitiation complex on both LTRs. Indeed, it has been demonstrated that the two HTLV-1 LTRs are functionally equivalent in HTLV-1-infected cell lines and ATL cells with a nearly equal distribution of transcription factors (CREB, ATF-1, c-Fos, c-Jun) and regulatory cofactors (p300/CBP, RNA polymerase II) [41]. Thus sense/antisense transcription is initiated by the same cellular and viral activators including CREB and Tax [29]. It is thus likely that sense transcription especially upon induction by the viral trans-activator Tax contributes in keeping antisense transcription to low levels. On the other hand, when Tax expression is disrupted by different mechanisms including down-regulation of its expression by accessory viral proteins (like Rex and p30) or by hypermethylation of the 5′ LTR, antisense transcription is thereby augmented. Data collected from different infected cells have also provided important information as to the process of HTLV-1 gene expression. By real-time RT-PCR, quantification of HBZ and Tax mRNA levels confirmed that primary ATL cells expressed high levels of HBZ but low levels of Tax mRNA. This pattern was distinguishable from that of infected T- cell lines, showing low HBZ and high Tax mRNA levels [17]. However, Tax has also been shown to induce cellular senescence [42]. The decline of Tax expression may be a result of the loss of cells that express high levels of Tax. Only cells that express low levels of Tax and high levels of HBZ could undergo mitotic expansion.

Recent data using the *in vivo* rabbit model have further reinforced the notion that antisense transcription is maintained over time unlike sense transcription. Kinetic analysis revealed that sense transcription was expressed at the highest levels immediately after infection and then progressively declined over time [43]. Conversely, antisense transcription was expressed at a low level early after infection and continued to increase before reaching a plateau. These results confirm an inverse correlation between sense/antisense transcription and Tax/HBZ expression over time, which provided important evidence linking HBZ expression to the survival of the infected cells in the host. Moreover, very recent results show that an efficient HBZ-specific CD8^+^ T-cell response reduced the proviral load and the risk of HAM/TSP [44], confirming that the anti-HBZ response constitutes an efficient protection against the proliferation of HTLV-1-infected cells. On the other hand, the same HBZ-specific cytotoxic T-lymphocyte clone was unable to lyse leukemic cells isolated from a patient with ATL [45]. Further clarification of the mechanism involved in the resistance of ATL cells to the HBZ-specific T-cell response is needed to understand this observation.

## 5. Antisense Transcription and Other HTLVs

**Figure 4:**
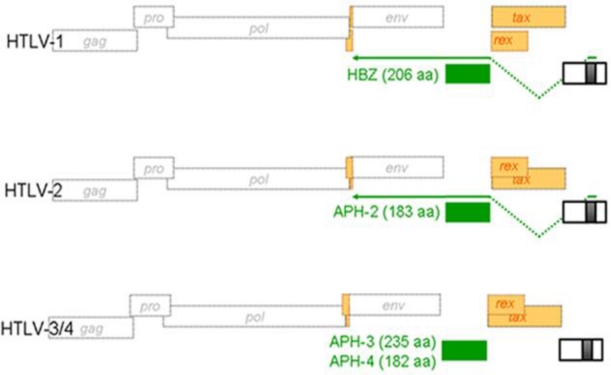
Schematic representation of antisense open reading frame (ORF) in other HTLV proviral genomes. Positioning of antisense transcript-encoded proteins termed APH in HTLV-2, HTLV-3 and HTLV-4 is similar to HBZ in HTLV-1. These proteins do share certain additional similarities with HBZ although their cellular localization and amino acid sequence do indicate functional differences. Recent results from our team have confirmed that both synthesis of APH-3 and APH-4 are dependent on a spliced transcript.

Unlike HTLV-1, the human HTLV-2 has been linked to HAM-like pathologies, but not to leukemia. As HBZ has been associated with ATL maintenance, our group has searched for a possible equivalent antisense transcript in HTLV-2. We have indeed reported that HTLV-2 produced an antisense spliced transcript, which initiated in the 3′ LTR and encoded for a protein that we have termed Antisense Protein of HTLV-2 (APH-2) (Figure 4) [46]. Distinctively from HBZ, APH-2 does not possess a bZIP consensus domain but retained the capacity to inhibit Tax2-dependent LTR activation and to interact with the CREB transcription factor. Furthermore, unlike HBZ, APH-2 did not co-localize with the nucleolus, although in non-T and T cells, it did demonstrate a nuclear localization. These results have indicated that HTLV-1 is not a unique retrovirus in its capacity to produce encoding antisense transcripts. They also raised the intriguing possibility that differences in protein domains and in nuclear distribution suggest potential dissimilarities in their capacity to modulate cellular and/or viral expression. These functional differences could eventually provide an explanation for the inability of HTLV-2 to cause leukemia in infected patients. Further studies will be required to determine how HBZ and APH-2 vary in their impact on cellular gene expression.

We have also recently tested whether the newly discovered human retroviruses HTLV-3 and HTLV-4 [47,48] were equally capable of producing an antisense transcript. Indeed, our results have indicated that both viruses produce a spliced and polyadenylated antisense transcript. The encoded proteins showed distinct localization; the HTLV-3 antisense protein being both nuclear and cytoplasmic, while the HTLV-4 counterpart being almost exclusively contained in the nucleus (Larocque et al., unpublished results). In addition, alike APH-2, both APH-3 and APH-4 lacked a consensus bZIP domain but did block LTR activation mediated by their respective Tax protein. Thus, cellular localization is distinguishing these various antisense transcript-encoded retroviral proteins and could suggest that they functional affect infected cells in a different manner. We are currently looking at how these proteins alter activation of transcription factors known to be functionally modulated by HBZ. Functional comparison of these viral proteins should provide important information as to their role in viral replication and alteration at the cellular level.

## 6. Summary and Conclusion

Since the discovery of HBZ in 2002, accumulating evidence suggests that the development of HTLV-1 infection requires the *Tax* and *HBZ* genes, whose expression is respectively controlled by the 5′ and 3′ LTR. In the first stage of the infection of T cells by HTLV-1, *Tax* expression is high and through a positive feedback stimulates protein viral synthesis, virus production, and viral infection. However, cells highly expressing HTLV-1 proteins are eliminated by the humoral response and CTL activity of the host. At this stage, HBZ can play a crucial role by down-regulating the 5′-LTR-dependent viral transcription and may allow infected cells to evade the immune response. In additionally, HBZ promotes the proliferation of infected T lymphocytes. This dual action probably confers a survival advantage on HBZ-expressing cells and is consistent with the observation that HBZ favors the establishment of persistent infection in HTLV-1-inoculated rabbits [24] and induces T-cell lymphoma and systemic inflammation [36]. Henceforth, it is clear that interfering with HBZ function may be a useful strategy for the treatment of ATL and HAM/TSP [49]. Future studies will also permit to determine whether APH-2 also plays an essential role in the development of HTLV-2-associated diseases.
